# In Vivo Cardiac Cellular Reprogramming Efficacy Is Enhanced by Angiogenic Preconditioning of the Infarcted Myocardium With Vascular Endothelial Growth Factor

**DOI:** 10.1161/JAHA.112.005652

**Published:** 2012-12-19

**Authors:** Megumi Mathison, Robert P. Gersch, Ahmed Nasser, Sarit Lilo, Mallory Korman, Mitchell Fourman, Neil Hackett, Kenneth Shroyer, Jianchang Yang, Yupo Ma, Ronald G. Crystal, Todd K. Rosengart

**Affiliations:** 1 Department of Surgery, Stony Brook University Medical Center, Stony Brook, NY (M.M., R.G., A.N., S.L., M.K., M.F., J.Y., T.K.R.); 2 Department of Pathology, Stony Brook University Medical Center, Stony Brook, NY (M.K., K.S., J.Y., Y.M.); 3 Department of Genetic Medicine, Weill Cornell Medical College, New York, NY (N.H., R.G.C.)

**Keywords:** angiogenesis, gene therapy, myocardial infarction, stem cells

## Abstract

**Background:**

In situ cellular reprogramming offers the possibility of regenerating functional cardiomyocytes directly from scar fibroblasts, obviating the challenges of cell implantation. We hypothesized that pretreating scar with gene transfer of the angiogenic vascular endothelial growth factor (VEGF) would enhance the efficacy of this strategy.

**Methods and Results:**

Gata4, Mef2c, and Tbx5 (GMT) administration via lentiviral transduction was demonstrated to transdifferentiate rat fibroblasts into (induced) cardiomyocytes in vitro by cardiomyocyte marker studies. Fisher 344 rats underwent coronary ligation and intramyocardial administration of an adenovirus encoding all 3 major isoforms of VEGF (AdVEGF‐All6A^+^) or an AdNull control vector (n=12/group). Lentivirus encoding GMT or a GFP control was administered to each animal 3 weeks later, followed by histologic and echocardiographic analyses. GMT administration reduced the extent of fibrosis by half compared with GFP controls (12±2% vs 24±3%, *P*<0.01) and reduced the number of myofibroblasts detected in the infarct zone by 4‐fold. GMT‐treated animals also demonstrated greater density of cardiomyocyte‐specific marker beta myosin heavy chain 7^+^ cells compared with animals receiving GFP with or without VEGF (*P*<0.01). Ejection fraction was significantly improved after GMT vs GFP administration (12±3% vs −7±3%, *P*<0.01). Eight (73%) GFP animals but no GMT animals demonstrated decreased ejection fraction during this interval (*P*<0.01). Also, improvement in ejection fraction was 4‐fold greater in GMT/VEGF vs GMT/null animals (17±2% vs 4±1%, *P*<0.05).

**Conclusions:**

VEGF administration to infarcted myocardium enhances the efficacy of GMT‐mediated cellular reprogramming in improving myocardial function and reducing the extent of myocardial fibrosis compared with the use of GMT or VEGF alone.

## Introduction

Coronary artery disease (CAD) remains the leading cause of death in the west, in part because of the still limited options for the treatment of diffuse CAD, myocardial infarction, and congestive heart failure.^1–4^ Cardiac stem cell therapy has been embraced as a new approach to treating end‐stage heart disease that theoretically repopulates otherwise permanently scarred myocardium with contractile cells.^5–13^ The creation of induced pluripotent stem cells (iPSCs) and the generation of cardiomyocyte‐like cells from iPSCs appear to have represented breakthroughs in this field,^14–23^ but recent reports of iPSC tumorogenicity and immunogenicity may ultimately reflect limits of the clinical applicability of iPSCs, as may the logistic challenges of iPSC delivery in the clinical setting.^24–27^

The recent discovery by Ieda et al, as confirmed by others, that a trio of “cardio‐differentiating” transcription factors could be used to generate “induced cardiomyocyte” (iCM) cells *directly* from somatic cells offers the exciting new possibility of generating autologous cells that possess characteristics that are *at least* consistent with those of a cardiomyocyte phenotype production.^28–35^ Perhaps more important, this novel regenerative strategy offers the intriguing potential to bypass iPSC staging *and* convert myocardial scar fibroblasts into functional iCMs in situ, potentially transforming regions of myocardial infarction back into functioning myocardium.^30,32–34^

In the context of conflicting recent reports regarding the potential efficacy of such in situ myocardial regeneration,^30,32–36^ we hypothesized that ischemia could adversely affect the survival and/or function of iCMs in the infarct zone, much as it causes the loss of native cardiomyocytes and exogenous (stem cell) implants.^37–41^ In this scenario, adequate myocardial scar vascularization would be an important component of an optimized in situ cellular reprogramming strategy.^40–41^ We now accordingly report that the (lentivirus‐mediated) administration of a cocktail of 3 transcription factors (Gata4, Mef2c, and Tbx5 [GMT]), used as a stimulus for iCM generation together with administration of an adenovirus encoding all 3 major isoforms of vascular endothelial growth factor (VEGF), results in greater improvements in postinfarct myocardial function than does the administration of GMT or VEGF alone.

## Methods

### Vectors and Cells

An adenovirus vector (AdVEGF‐All6A^+^) based on an Ad5 serotype backbone with deletions in the E1 and E3 regions and containing an artificial splice sequence cassette was used to provide delivery of all 3 major isoforms of VEGF (121, 165, and 189).^42^ An analogous construct with an empty expression cassette (AdNull) was used as a control vector.

Lentivirus vectors were constructed to provide expression of GMT in targeted myocardial tissues. For GMT vector construction, RNA was first isolated from rat heart using TRIzol reagent (Invitrogen) and converted to cDNA using a reverse transcription kit (Roche). These samples (for Mef2c) or commercially available cDNA (for Gata4 and Tbx5; AddGene) were used to amplify relevant coding sequences using primers based on the PubMed nucleotide coding sequences for these 3 transgenes (Table 1).^28,30^ Primers were designed to include an *Sal*1 binding site 5′ and *Xho*1 restriction site 3′ of each coding sequence. Gene inserts were amplified using a polymerase chain reaction kit (Roche) and cloned into the pENTR3C vector before homologous recombination into the F12‐CMV vector (Invitrogen) under the control of a cytomegalovirus promoter. F12‐CMV also includes an eGFP cassette under the control of an ubiquitin promoter for lineage and efficiency analysis.

**Table 1.** Primers Used for Plasmid Construction

Primer Name	Sequence	Melting Temperature
Gata4 5′	GGCGGTCGACATGTACCAAAGCCTGGCTATG	72.6
Gata4 3′	GATTCTCGAGTACGCGGTGATTATGTCCCCATG	70.9
Mef2c 5′	GGACTGTCGACATGGGGAGAAAAAAGATTCAG	68.5
Mef2c 3′	TGTGACTCGAGTCATGTTGCCCATCCTTCAGAGAG	71.7
Tbx5 5′	CACCGTCGACATGGCCGACGCAGATGAG	73.4
Tbx5 3′	CCTTCTCGAGTCAAGCTATTCTCGCTCCACTCTG	71.9

Gata4, Mef2c, and Tbx5 F12‐CMV plasmids thus generated were transfected into the 293T human kidney fibroblasts cell line (ATCC) along with gateway system using plasmids pMD2G and psPAX via Lipofetamine 2000 reagent (Invitrogen). Viral bearing supernatant was isolated and cellular debris was removed by centrifugation and syringe filtering (0.45‐μm pore size; Sarstedt). Virus was further concentrated by centrifugation (2 hours at 10 000*g*), and supernatants were then aspirated and pellets diluted in viral diluent (3% sucrose, 10 mmol/L Tris‐HCl, pH 7.6, 150 mmol/L NaCl).

Dermal fibroblasts derived from 1‐cm^2^ biopsy samples of the abdominal skin of Fisher 344 rats were plated onto plastic dishes.^18,28^ Attached fibroblasts were cultured for 7 days in DMEM/10% FBS at 10^4^/cm^2^. Cells were then replated and infected with appropriate vectors after 24 hours. Cardiomyocytes were obtained from the neonatal ventricles of Sprague Dawley rats (gift of E. Entcheva, Stony Brook University, Stony Brook, NY), which were cut into small pieces and digested with collagenase type II solution. A single‐cell suspension of primary cardiomyocytes was then obtained by gentle passage through a 40‐μm cell strainer, and cells were plated onto tissue culture–treated dishes (Thermo Fisher Scientific).

### In Vitro Immunofluorescence Studies

Lentivirus encoding GMT or GFP control vectors in the presence of 8 μg/mL polybrene (Millipore) was added to rat dermal fibroblast culture media (DMEM+10% FBS) for 16 hours. This medium was then removed and the cells were allowed to transdifferentiate under normal culture conditions over a 14‐day course. These cells were washed twice with PBS and fixed with 4% paraformaldehyde (Affymetrix) for 10 minutes. Cells were then permeabilized with 0.5% Saponin (Sigma) at room temperature for 10 minutes. Slides were blocked with 10% goat serum (Santa Cruz Technology) before incubation in 5% goat serum with primary antibodies directed toward cardiac troponin T (Abcam), beta myosin heavy chain 7 (MYH7; Sigma), or α‐sarcomeric actinin (Sigma). Primary antibodies were bound with fluorescent secondary (647 nm; Alexa Fluor, Invitrogen), and fluorescence was visualized using a Ti‐S inverted phase/fluorescent microscope with SPOT cooled 2.0‐megapixel digital camera system (Nikon).

### Fluorescence Activated Cell Sorting

For fluorescence activated cell sorting analyses, cells were washed with PBS and treated with 0.05% trypsin (Gibco). These cells were then pelleted and permeabilized with 0.1% Saponin for 30 minutes at 4°C, repelleted and suspended in 5% goat serum, and incubated with relevant primary antibodies followed by incubation with a fluorescent secondary antibody (647 nm; Alexa Fluor). Cells were fixed with 1% paraformaldehyde and analyzed for fluorescence using Cell Quest V3.3 software on an FACSCalibur Flow Cytometer (Becton, Dickinson, and Co).^28–30^

### Myocardial Infarction Animal Model

Myocardial infarction was created by coronary ligation, as previously described, in adult male Fisher 344 rats (275 to 300 g; Harlan, Indianapolis, Ind), using protocols approved by the State University of New York, Stony Brook Institutional Animal Care and Use Committee.^40–41^ Animals were housed, operated on, and cared for in facilities run by the Division of Laboratory Animal Resources at Stony Brook University, which is fully accredited by the Association for the Assessment and Accreditation of Laboratory Animal Care International.

Animals were first anesthetized with isofluorane 4% in an induction box, intubated, and placed on a rodent ventilator (Harvard Apparatus) using isofluorane inhalation (3.5%) supplemented with oxygen. Lidocaine (4 mg/kg) was administered intramuscularly. A left thoracotomy was then performed, and the left coronary artery was ligated 1 to 2 mm from its origin with a 7‐0 polypropylene suture. This prep consistently produces a gross (pale) anteroapical myocardial infarction with <20% mortality.

At the time of coronary ligation, 5 uniformly distributed 20‐μL injections, each containing 2×10^8^ particle units (1×10^9^ total dose) of Ad VEGF‐All6A^+^ or AdNull (n=12/group), were administered around the infarct zone, identified as an area of blanching on the anterolateral wall of the left ventricle, by operators blinded to treatment group. The chest was then sutured closed in a layer‐by‐layer manner, and the animals were placed in a heated chamber and allowed to recover under supervision. Ketorolac (3 to 5 mg/kg) and buprenorphine (0.05 to 0.1 mg/kg) were administered subcutaneously at the time of closure and every 12 to 24 hours postoperatively as needed, determined by the level of activity displayed by the animals.

A second thoracotomy was performed 3 weeks later, and animals previously receiving AdVEGF‐All6A^+^ or AdNull each then underwent administration into the infarct zone of 1×10^5^ transducing units of lentivirus (5 uniformly distributed 20‐μL injections) encoding Gata4, Mef2c, or Tbx5 coupled to a GFP marker or encoding GFP alone (final n=6/group). Animal were again recovered as described earlier. Euthanasia was later achieved 4 weeks later by deep (4%) isoflurane anesthesia followed by exsanguination, consistent with American Veterinary Medical Association guidelines.

### Echocardiography

Echocardiography was performed under light anesthesia with 3% isoflurane using a Veno 770 Imaging System (VisualSonics Inc., Toronto, Ontario, Canada) at 5 different time points: before and 3 days after coronary ligation and AdVEGF‐All6A^+^ or AdNull, at the time of GMT or GFP administration 21 days later (baseline), and then 2 weeks and 4 weeks later after baseline (postligation day 35 and day 49, respectively).^40–41^ Echo images were obtained of the left ventricle in both parasternal long‐axis and short‐axis views by investigators blinded to treatment group. Left ventricular end‐systolic and end‐diastolic diameters and left ventricular septal and posterior thickness (in both end‐systolic and end‐diastolic phases) were measured from M‐mode tracings. These imaging data were then analyzed by investigators blinded to treatment group. Change in ejection fraction (EF) was calculated as: (EF at day 49)−(EF at day 21)]/(EF at day 21).

### Histologic Analyses

To obtain cardiac tissue specimens, animals were exsanguinated under deep anesthesia via an incision made in the right atrium. While the heart was still beating, it was perfused with normal saline and fixed with PBS (pH 7.2) containing 4% (w/v) paraformaldehyde via a 25‐gauge needle inserted into the left ventricular apex. The heart was then harvested and rinsed with saline to clear the blood. Excised hearts were fixed with 4% paraformaldehyde for 24 hours and then 2% paraformaldehyde for 48 hours at 4°C. The heart was then cut transversally and sectioned with 2 (2‐ to 3‐mm) slices obtained, 1 immediately cephalad and 1 immediately caudad to the transverse centerline of the infarct region, which was readily identifiable by gross inspection. After paraffin embedding of these slices, seven 5‐μm‐thick sections were obtained at 90‐μm intervals.

**Figure 1.**
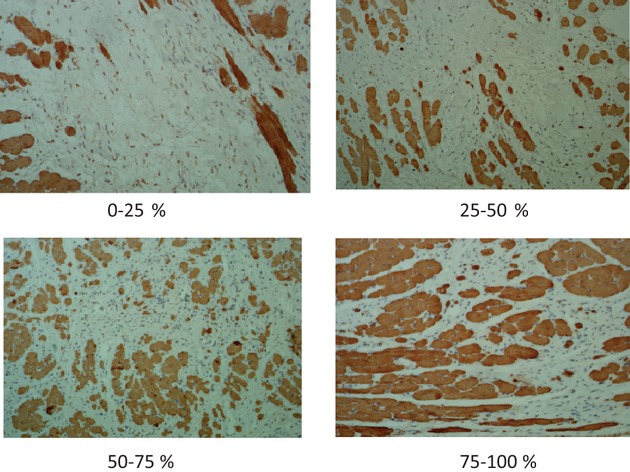
MHY7 cell density classification. MYH7^+^ cell number was analyzed in a semiquantitative manner as the MYH7^+^ cell density in 5 areas per slide viewed at ×200 magnification (at the center of the infarction zone, in the mid areas between the center of infarction and the border zone, and in each border zone adjacent to the infarct). These fields were graded by an investigator blinded to treatment group as follows: (top left) grade I: <25% of selected microscopic field demonstrating MYH7^+^ cells; (top right) grade II: 25% to 50% of selected microscopic field demonstrating MYH7^+^ cells; (bottom left) grade III: 50% to 75% of selected microscopic field demonstrating MYH7^+^ cells; and (bottom right) grade IV: >75% of selected microscopic field demonstrating MYH7^+^ cells. MYH7 indicates myosin heavy chain 7.

For analyses of in vivo cellular reprogramming, microscopic slides of every other section obtained as described were stained with primary antibodies against beta myosin heavy chain 7 (anti‐MYH7, Sigma) and then incubated with secondary IgG antibody. Five microscopic fields per slide (at the center of the infarction zone, in the mid areas between the center of infarction and the border zone [left and right], and in each border zone adjacent to the infarct [left and right]) viewed at ×200 magnification were graded semiquantitatively to determine MYH7^+^ cell density. Density grading assessed by an investigator blinded to treatment group were defined as follows: grade I: <25% of selected microscopic field demonstrating MYH7^+^ cells; grade II: 25% to 50% of selected microscopic field demonstrating MYH7^+^ cells; grade III: 50% to 75% of selected microscopic field demonstrating MYH7^+^ cells; and grade IV: >75% of selected microscopic field demonstrating MYH7^+^ cells (Figure 1). These observations are reported as the percentage of fields per animal demonstrating a given density grade and the mean of these percentages per group for all animals with at least 10 fields analyzable within an infarct zone.

To assess the extent of fibrosis, 22 sections per animal (at a 120‐μm interval between each section) obtained as described earlier were stained with Masson's trichrome. The fibrotic area (blue) and the nonfibrotic region (red) were outlined using Adobe Photoshop CS5 software, and then quantified with MATLAB and Simulink software (MathWorks, Inc). The total area of fibrosis was calculated as: (total of blue pixels from all sections/total of blue plus red pixels from all sections).

For myofibroblast identification, 2 sections per animal demonstrating the greatest cross‐sectional area of fibrosis, as determined by trichrome staining, were stained for α‐smooth muscle actin (α‐SMA; Anti‐Actin‐Smooth Muscle, Spring Bioscience). α‐SMA–positive cells in these sections exclusive of those found in vascular structures or endocardium were counted at ×200 magnification.

For vascularization studies, the number of vessels per microscopic field was determined from the sections stained as described with α‐SMA or with factor VIII (anti–factor VIII–related antigen, Ventana) and counted at ×200 or ×400, respectively.

### Statistical Analysis

Statistical analysis was performed with SAS version 9.2. The data are presented as mean±SEM. The normality of the data was examined with Shapiro–Wilk. When there was a normal distribution, an ANOVA was performed to detect statistical significances between multiple groups. When the ANOVA showed significance, a Student *t* test was performed with a post hoc Holm–Bonferroni correction. When there was not a normal distribution, a Kruskal–Wallis test was performed. When the test was significant, the Wilcoxon rank test was performed with a post hoc Holm‐Bonferroni correction. For categorical variables, a Fisher exact test was performed. Values of *P*<0.05 were considered statistically significant.

## Results

### In Vitro iCM Generation

**Figure 2.**
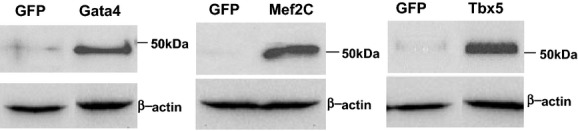
Generation of lentivirus encoding individual cardiac transcription factors. Lentivirus encoding the transcription factors Gata4, Mef2c, and Tbx5 were prepared in 293T package cells as described in Methods and Results and protein expression was detected by Western blot analysis using antibodies specific to each transgene. A lentivirus expressing a GFP marker gene was used as a control for these expression vectors and β‐actin was used as a loading control. GFP indicates green fluorescent protein.

**Figure 3.**
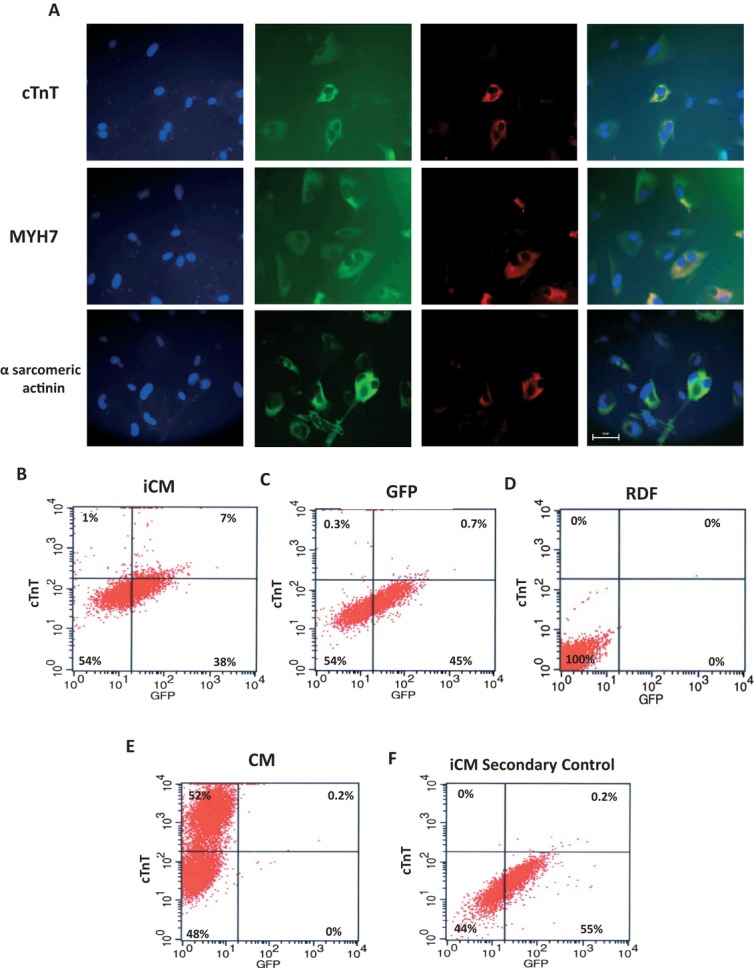
iCM generation in vitro. Primary rat dermal fibroblast (RDF) cells were cultured and infected with GMT or a GFP control lentivirus as described in Methods and Results. Fourteen days after infection, cells were fixed and stained for specified cardiomyocyte markers. A, Immunofluorescence studies. First column represents 4',6‐diamidino‐2‐phenylindole (DAPI) staining to identify cell nuclei. Second column represents GFP fluorescence to identify cells infected by at least one of the lentivirus vectors. Third column represents red staining of relevant cardiomyocyte markers (first row: cardiac troponin T [cTnT]; second row: myosin heavy chain 7 [MHY7]; third row: α‐sarcomeric actinin). Fourth column depicts merge of previous 3 images. Note coincidence of these respective markers and binucleated cells typical of cardiomyocyte, and that GFP (−) cells also fail to express markers. Uninfected RDFs did not express either marker (not shown). All photomicrographs were taken at ×400 magnification (bar=50 μm). B through F, FACS analysis. Depicted are FACS plots for cTnT staining after: (B) RDFs infected with GMT, demonstrating 7% expression of cTnT in GFP^+^ cells, (C) RDFs infected with GFP control lentivirus, (D) uninfected RDFs, (E) primary cardiomyocyte control, and (F) RDFs infected with GMT, with use of secondary antibody only. Graphs show a minimum of 5000 events. iCM indicates induced cardiomyocytes; GMT, Gata4, Mef 2c, and Tbx5; FACS, fluorescence activated cell sorting.

The competency of each of the GMT lentivirus vectors was confirmed by in vitro cell infection assays, which demonstrated expression of all 3 of the reprogramming transcription factors (Figure 2). Confirmation of the ability of these transcription factors to induce iCM transdifferentiation was obtained by immunofluorescence staining of rat dermal fibroblasts after GMT lentiviral transduction. In these studies, cells exposed to GMT vectors expressed cardiomyocyte‐specific markers including cardiac troponin T, α‐sarcomeric actin in, and MYH7, while uninfected cells and fibroblasts exposed only to a GFP control vector failed to express these markers (Figure 3A). On fluorescence activated cell sorting quantification, GMT infection demonstrated evidence of cardiomyocyte marker expression by ≈7% of infected fibroblasts (Figure 3B through 3F).

### Vascularization of Infarcted Myocardium

**Figure 4.**
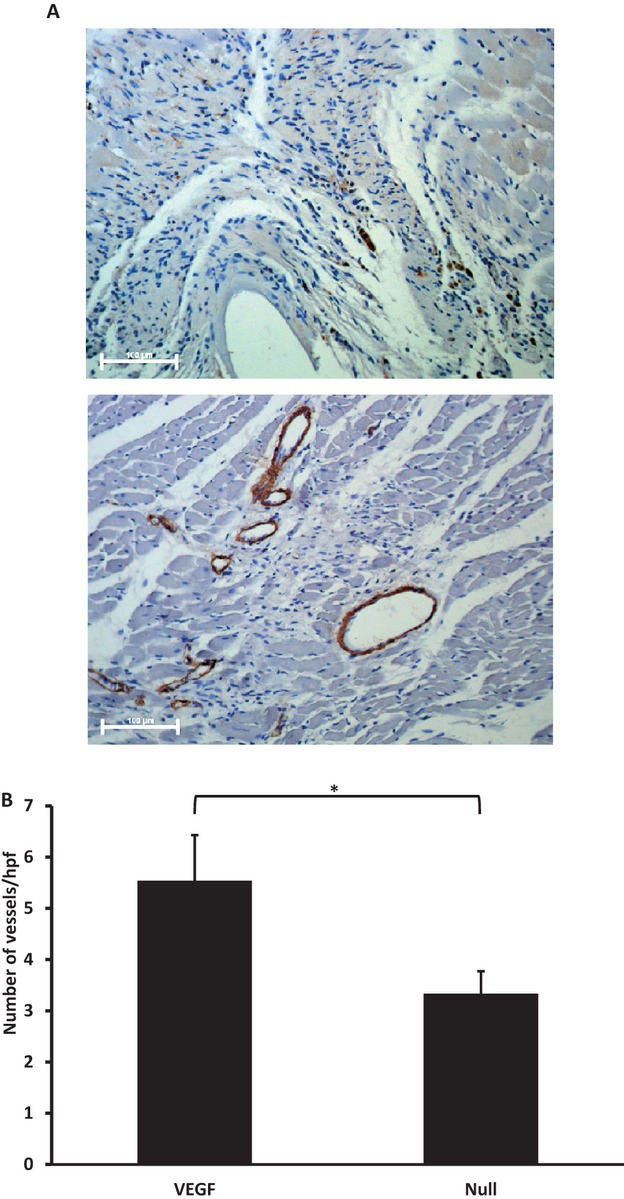
Myocardial vascularization studies. Vascularization of infarct regions assessed by staining for α‐smooth muscle actin in sections obtained as described in Methods and Results 7 weeks after coronary ligation and administration of AdVEGF‐All6A^+^ or the control vector AdNull. A, Photomicrographs of representative sections of infarct zones viewed at ×200 after administration of AdNull/GMT (top) or AdVEGF‐All6A^+^/GMT (bottom). Bars represent 100 μm. B, Quantification of vascularization of infarct regions as assessed by the number of vessel per high power (×200) microscopic field (n=12/group). *P<0.05. VEGF indicates vascular endothelial growth factor; GMT, Gata4, Mef2c, and Tbx5.

Vascularization of the infarct region as assessed by α‐SMA staining was significantly greater in AdVEGF‐All6A^+^–treated animals than in animals receiving an AdNull control vector: 5.5±0.9 vs 3.3±0.4, respectively; *P*<0.05 (Figure 4). A similar, approximate 2‐fold increase in vessels stained with factor VIII was noted in animals receiving AdVEGF‐All6A^+^ alone vs those receiving AdNull alone (14.1±2.1 vs 7.3±1.4, *P*<0.05).

### Histologic Assessment of Postinfarct Ventricles

**Figure 5.**
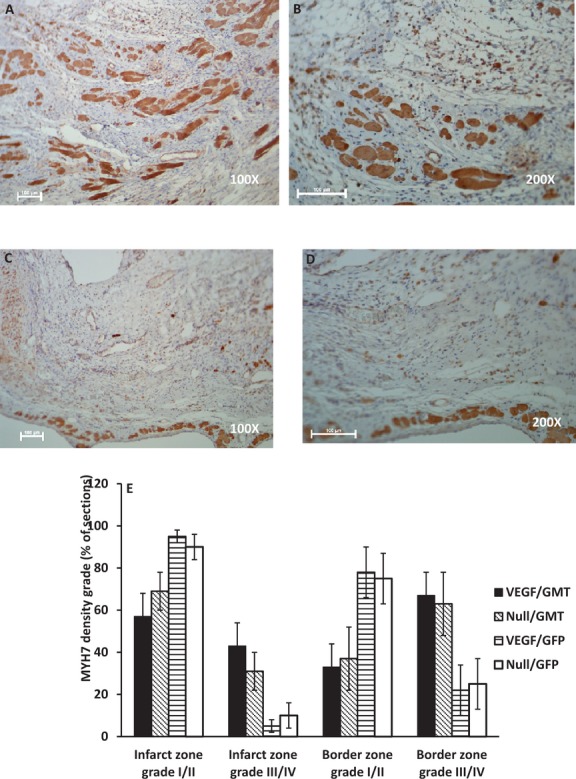
Cardiomyocyte density in infarct zones. Cardiomyocyte‐specific marker MYH7 staining of the infarct and border zones of sections of myocardium harvested 7 weeks after coronary ligation and administration of AdVEGF‐All6A^+^ or the control vector AdNull (4 weeks after the administration of lentivirus encoding GMT or a GFP control). A through D, Photomicrographs of representative sections of infarct zones from animals treated with AdVEGF‐All6A^+^/GMT (top row) or/AdNull/GFP (bottom row) at ×100 (left) and ×200 (right) magnification, respectively. Bars represent 100 μm. E, Depiction of MYH7 cell density as a percent of total sections analyzed (n=6/group). Grade I/II indicates than <50% of the examined microscopic fields were occupied by MYH7^+^ cells; grade III/IV indicates than >50% of the examined microscopic fields were occupied by MYH7^+^ cells (see Methods and Results for definitions and Figure 1 for microscopic fields representative of each density grade). MYH7 indicates myosin heavy chain 7; VEGF, vascular endothelial growth factor; GMT, Gata4, Mef2c, and Tbx5; GFP, green fluorescent protein.

To assess the efficacy of GMT administration with or without VEGF pretreatment in vivo, a series of histologic analyses were performed on sections of the heart obtained 4 weeks after GMT/GFP delivery (7 weeks after coronary ligation and AdVEGF‐All6A^+^/AdNull administration). These studies demonstrated a greater density of cells staining for the cardiomyocyte marker MYH7 in the infarct zone in GMT‐treated animals compared with control animals (Figure 5A through 5D). Typically, GMT‐treated animals demonstrated relatively large islands of MYH7^+^ cells compared with sparse foci of MYH7^+^ in control animals.

Using a grade I to IV scale to semiquantitatively assess MYH7 cell density, GMT‐treated animals demonstrated grade III/IV MYH7 cell density in the infarct zone in a greater percentage of microscopic fields than did GFP control animals, in both the infarct zone and border zones (Table 2). The percentage of microscopic fields demonstrating grade III/IV MYH7 cell density was significantly greater in GMT‐treated animals compared with GFP‐treated control animals (36±8% vs 7±4%, *P*<0.01) in the infarct zone and in the border zones adjacent to infarct zones (65±10% vs 23±9%, *P*<0.01). Notably, none of the sections demonstrated grade IV MYH7 cell density in the infarct zone in the GFP control groups (Table 2). Administration of AdVEGF‐All6A^+^ did not significantly alter MYH7 cell density (Figure 5E).

**Table 2.** MYH7^+^ Cell Density*

	Grade I*	Grade II*	Grade III*	Grade IV*
Infarction area				
AdVEGF‐All6A^+^/GMT	12±5	44±8	30±7	14±5
AdNull/GMT	29±13	41±8	22±7	8±5
AdVEGF‐All6A^+^/GFP	61±14	34±13	5±3	0
AdNull/GFP	50±14	40±10	10±6	0
Border zone area				
AdVEGF‐All6A^+^/GMT	4±3	29±9	48±10	19±7
AdNull/GMT	14±10	24±7	38±9	25±10
AdVEGF‐All6A^+^/GFP	30±14	48±11	21±12	1±1
AdNull/GFP	43±13	32±7	22±10	3±2

MYH7 indicates myosin heavy chain 7; VEGF, vascular endothelial growth factor; GMT, Gata4, Mef2c, and Tbx5.

* Percentage of microscopic fields analyzed demonstrating density grade (n=6 animals per treatment group).

* Grade I: <25% of microscopic field containing MYH7^+^ cells; grade II: 25% to 50% of microscopic field containing MYH7^+^ cells; grade III: 50% to 75% of microscopic field containing MYH7^+^ cells; grade IV: >75% of microscopic field containing MYH7^+^ cells. All measurements at ×200.

**Figure 6.**
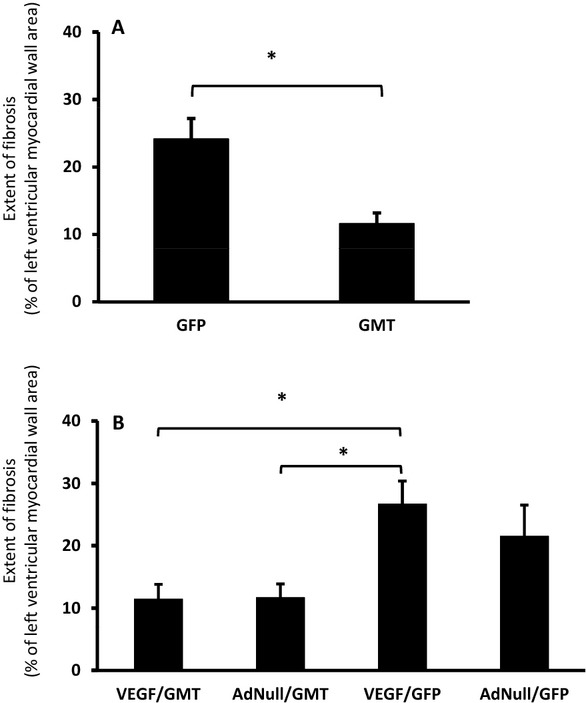
Extent of left ventricular wall fibrosis. The percent of left ventricular myocardial wall area demonstrating fibrosis as determined by trichrome staining of sections of myocardial tissue harvested 7 weeks after coronary ligation and administration of AdVEGF‐All6A^+^ or the control vector AdNull (4 weeks after the administration of lentivirus encoding GMT or a GFP control) animals is depicted. A, Extent of fibrosis in animals receiving GMT versus GFP control vectors (n=12); *P<0.01. B, Extent of fibrosis for the 4 treatment groups (n=6/group); *P<0.05. GFP indicates green fluorescent protein; GMT, Gata4, Mef2c, and Tbx5; VEGF, vascular endothelial growth factor.

The extent of fibrosis in these sections, as detected by trichrome staining, was also significantly reduced in GMT‐treated animals compared with those receiving GFP, regardless of VEGF administration (Figure 6A). The cross‐sectional area of fibrosis in these groups, as a percentage of total left ventricular myocardial area in sections analyzed, was 12±2% vs 24±3% (*P*<0.01). No difference in the extent of fibrosis was detected in animals treated with AdVEGF‐All6A^+^ without GMT compared with AdNull/GFP controls (Figure 6B). Also, AdVEGF‐All6A^+^ administration did not further reduce the extent of fibrosis compared with animals treated with GMT without VEGF.

**Figure 7.**
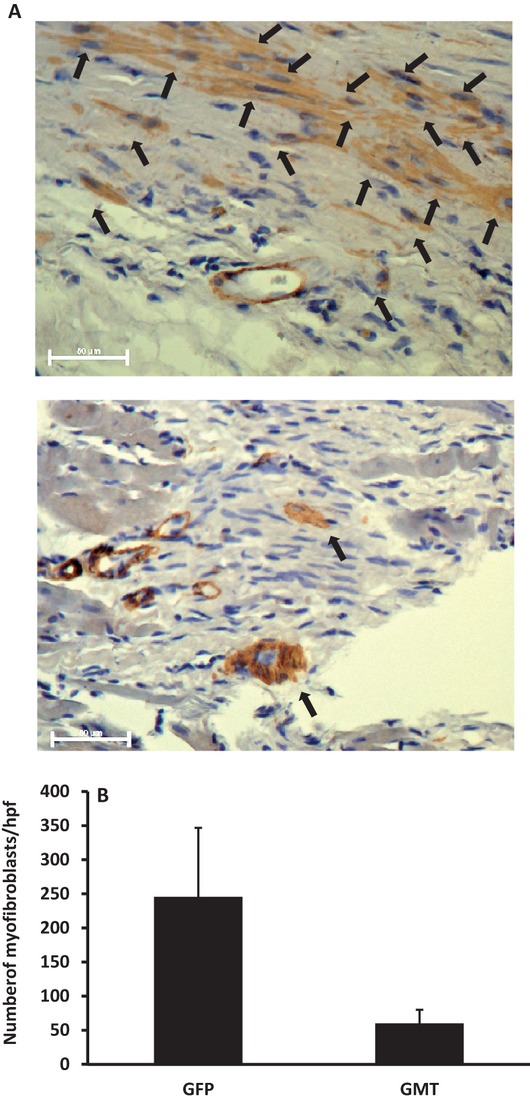
Myofibroblasts. Myofibroblasts identified by nonvascular α‐smooth muscle actin staining of the infarct and border zones of sections of myocardium harvested 7 weeks after coronary ligation and administration of AdVEGF‐All6A^+^ or the control vector AdNull (4 weeks after the administration of lentivirus encoding GMT or a GFP control). A, Photomicrographs of representative sections of infarct zones viewed at ×400 after administration of AdNull/GFP (top) or AdVEGF‐All6A^+^/GMT (bottom). Arrows depict cells identified as myofibroblasts. Bars represent 50 μm. B, The number of myofibroblasts identified per microscopic field (×200) in animals receiving GMT versus GFP control vectors (n=12), as identified by staining for α‐smooth muscle actin (*P*=0.09). GFP indicates green fluorescent protein; GMT, Gata4, Mef2c, and Tbx5; VEGF, vascular endothelial growth factor.

Consistent with the reduction in fibrosis detected in GMT‐treated animals, there was approximately a 4‐fold decrease in the number of myofibroblasts observed in GMT‐treated animals compared with control animals, regardless of VEGF administration (Figure 7).

### Improvement in Ventricular Function After GMT and VEGF Administration

**Figure 8.**
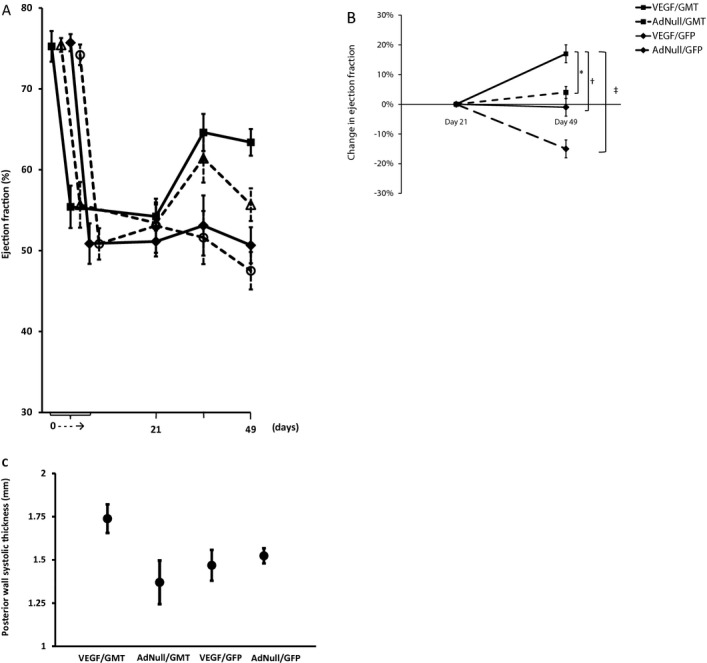
Echocardiographic analysis of global ventricular function after in vivo administration of cellular reprogramming and/or AdVEGF‐All6A^+^ vectors. Echocardiographic studies were performed at the specified time points before and after coronary ligation and administration of AdVEGF‐All6A^+^ or the control vector AdNull at time 0, and after the administration of lentivirus encoding GMT or a GFP control 3 weeks later (n=6). A, Global ejection fraction for each treatment group. At day 49 (Kruskal–Wallis rank test; *P*<0.005): AdVEGF‐All6A^+^/GMT versus AdNull/GFP:* P*<0.05; AdVEGF‐All6A^+^/GMT versus AdVEGF‐All6A^+^/GFP:* P*<0.05; AdVEGF‐All6A^+^/GMT versus AdNull/GMT:* P*=0.08; AdVEGF‐All6A^+^/GFP versus AdNull/GFP:* P*=0.86). B, Change in ejection fraction from the time of the lentivirus administration (day 21) baseline to the time of follow up echo 4 weeks later (day 49). Top panel: animals receiving GMT versus GFP control vector (n=12); *P<0.01. Bottom panel: each study group analyzed separately (n=6). *P=0.02; ^†^*P*=0.008; ^‡^*P*<0.001. C, Left ventricular posterior wall function. Left ventricular posterior wall thickness at end‐systole 7 weeks after coronary ligation and administration of AdVEGF‐All6A^+^ or the control vector AdNull (4 weeks after the administration of lentivirus encoding GMT or a GFP control). Differences between groups did not reach statistical significance (*P*=0.09). VEGF indicates vascular endothelial growth factor; GMT, Gata4, Mef2c, and Tbx5; MYH7, myosin heavy chain 7.

Echocardiography was performed to assess the functional implications of the outcomes noted above. Echo analyses performed before and immediately after coronary ligation demonstrated that EF was reduced by ≈30% from baseline values (Figure 8A). This decrease in cardiac function persisted 3 weeks later, at the time of GMT or GFP lentivirus administration.

As detailed in Table 3, mean EF 4 weeks after GMT or GFP administration was greatest for animals receiving GMT administration with AdVEGF‐All6A^+^ pretreatment (AdVEGF‐All6A^+^/GMT), being significantly greater than animals receiving either AdVEGF‐All6A^+^ without GMT (AdVEGF‐All6A^+^/GFP; *P*<0.05) or both control vectors (AdNull/GFP; *P*<0.05). Similar observations were made for differences between groups in fractional shortening (Table 4). When grouped together regardless of prior AdVEGF administration, GMT‐treated animals demonstrated significantly greater mean EF compared with similarly combined GFP control animals, both at 2 weeks and at 4 weeks after lentivirus administration (2 weeks: 63±2% vs 52±2%, *P*<0.01; 4 weeks: 60±2% vs 49±2%, *P*<0.001). In comparison, no difference in EF was seen after AdVEGF‐All6A^+^ administration alone (without GMT) vs animals receiving control vectors.

**Table 3.** Ejection Fraction Group Means as Assessed by Echocardiography*

Group	Time, d*				
	0	3	21	35	49
AdVEGF‐All6A^+^/GMT	75±2	55±3	54±2	65±2	63±2***
AdNull/GMT	75±1	56±3	53±2	61±3	56±2*
AdVEGF‐All6A^+^/GFP	76±1	51±3	51±2	53±4	51±2**
AdNull/GFP	74±1	51±2	53±3	52±3	48±2**

* All data expressed as a percentage ejection fraction (n=6 animals per treatment group). VEGF indicates vascular endothelial growth factor; GMT, Gata4, Mef2c, and Tbx5; MYH7, myosin heavy chain 7.

* Day 0 represents time of coronary ligation and AdVEGF‐All6A or AdNull administration; day 21 represents time of administration of lentivirus encoding GMT or GFP.

* AdVEGF‐All6A^+^/GMT versus AdNull/GFP at day 49: *P*<0.05.

* AdVEGF‐All6A^+^/GMT versus AdVEGF‐All6A^+^/GFP at day 49: *P*<0.05.

* AdVEGF‐All6A^+^/GMT versus AdNull/GMT at day 49: *P*=0.08.

* AdVEGF‐All6A^+^/GFP versus AdNull/GFP at day 49: *P*=0.86.

**Table 4.** Ventricular Functional Metrics as Assessed by Echocardiography*

Parameter	Time, d*				
	0	3	21	35	49
End‐diastolic volume*					
AdVEGF‐All6A^+^/GMT	217±7	181±13	215±17	222±21	234±25
AdNull/GMT	206±8	147±18	239±12	208±16	231±23
AdVEGF‐All6A^+^/GFP	192±11	197±11	302±30	240±32	280±32
AdNull/GFP	207±13	193±19	262±17	265±32	252±26
End‐systolic volume					
AdVEGF‐All6A^+^/GMT	54±5	80±7	99±10	80±11	84±7
AdNull/GMT	51±2	65±5	112±10	81±10	105±14
AdVEGF‐All6A^+^/GFP	47±3	97±8	148±17	117±23	139±20
AdNull/GFP	54±4	95±11	123±11	131±23	134±18
Fractional shortening					
AdVEGF‐All6A^+^/GMT	45±2	30±2	29±1	36±2	35±1***
AdNull/GMT	45±1	29±2	28±1	34±2	30±1**
AdVEGF‐All6A^+^/GFP	46±1	27±2	27±1	28±2	27±1*
AdNull/GFP	44±1	27±1	28±2	27±2	25±1**

* All data expressed as a percentage ejection fraction (n=6 animals per treatment group). VEGF indicates vascular endothelial growth factor; GMT, Gata4, Mef2c, and Tbx5; MYH7, myosin heavy chain 7.

* Day 0 represents time of coronary ligation and AdVEGF‐All6A or AdNull administration; day 21 represents time of administration of lentivirus encoding GMT or GFP.

* No significant difference between groups at any time interval.

* AdVEGF‐All6A+/GMT versus AdNull/GMT: *P*=0.08.

* AdVEGF‐All6A+/GMT versus AdVEGF‐All6A+/GFP: *P*<0.01.

* AdVEGF‐All6A+/GMT versus AdNull/GFP: *P*<0.0001.

* AdNull/GMT versus AdNull/GFP: *P*=0.07.

The change in EF from the time of the lentivirus administration baseline to the time of follow‐up echo 4 weeks later (Figure 8B, top) was also greater in the GMT vs GFP groups (12±3% vs −7±3%, *P*<0.01). Eight (73%) GFP‐treated animals but none of the GMT‐treated animals demonstrated *decreased* EF during this interval (*P*<0.01). Moreover, as depicted in Figure 8B (bottom), the improvement in EF observed in the AdVEGF‐All6A^+^/GMT group was 4 times greater than that observed for the AdNull/GMT group (17±2% vs 4±1%, *P*<0.05) and was significantly greater (*P*=0.008) than the change in EF observed after administration of VEGF alone (AdVEGF‐All6A^+^/GFP).

Interestingly, systolic wall thickening of the (remote, noninjected) left ventricular posterior wall also trended toward greater values in the GMT/VEGF group compared with animals receiving GMT without VEGF and compared with GFP/AdNull controls (Figure 8C).

## Discussion

The current study uses adenovirus‐mediated VEGF administration into infarcted myocardium to enhance the improvements in EF observed as early as 14 days after administration of the cellular reprogramming transcription factors Gata4, Mef2c, and Tbx5 alone.^30–33^ In addition to the novel incorporation of angiogenic preconditioning of the infarct milieu in this in situ myocardial regeneration strategy, the current work now also extends prior investigations by using a lentivirus vector to transfer GMT reprogramming factors in a second animal model, beyond the mouse model used to date.^28–35^ These current data stand in contradistinction to the recent work of other investigators, reported by Chen et al, suggesting that cellular reprogramming mediated by GMT strategy was ineffectual.^36^

Importantly, in situ cellular reprogramming offers the possibility of avoiding the challenges of current exogenous stem cell administration strategies, including the logistic barriers of cell procurement, expansion, and/or efficient delivery of such cells into a host myocardium.^31–34^ On the other hand, we now also demonstrate that angiogenic preconditioning of the infarcted myocardium enhances the efficacy of this strategy, extending prior observations with stem cell implants to this in situ strategy.^40–41^

The presumptive origin of improvements in postinfarct ventricular function in the current and prior cellular reprogramming studies is the generation of functional iCMs in areas of myocardial scar, as suggested by other investigators, and the enhancement of the survival and/or function of these “reprogrammed” myocytes by scar prevascularization.^30–33,30–41^ Interestingly though, our observations reinforce prior investigators' (previously undiscussed) observations of improvements in EF and decreases in fibrosis after cellular reprogramming that seem to far exceed what would be expected solely on the basis of the relatively inefficient generation of iCMs from substrate cells.^30–33^ More specifically, in comparison to a rate of transdifferentiating infarct fibroblasts into iCMs in vivo in the range of 1% to 20%, reductions in fibrosis and improvements in EF ranging up to 50% in the current and prior studies suggest alternative or additional mechanisms might be responsible for these outcomes.^30,32,34^

Several possible mechanisms, including ones related and unrelated to iCM generation, might explain these apparently disparate outcomes. First, it is theoretically possible that inconsistent coronary ligation technique and/or extent of infarction induced by ligation may be accountable for the differences in fibrosis/infarction observed in treated vs untreated animals. The development of significantly different treatment outcomes in the current study from baseline values that were not significantly different between groups argues against this possibility, as do the statistically significant *improvements* in function demonstrated between groups in the current study when using the baseline function of each animal as its own control. In the context of 2 previous studies that yielded similar outcomes, the larger sample size of these pooled data would seem to make it even less likely that these observations are attributable to an artifact due to coronary ligation technique.^30–33^

Assuming then that the observed differences in cardiac function are not artifactual, several causal mechanisms must be considered. The first simply implicates the generation of functionally competent, contractile iCMs that individually contribute to the restoration of global cardiac function. The significant increases in MYH7^+^ (cardiomyocyte) cell density in GMT‐treated vs control animals that we have observed in both the infarct and border zones, similar to the previous observations of Qian et al and Song et al, are supportive of this mechanism.^30,32^ Alternatively, the generation of iCMs in thinned zones of infarction might improve wall stresses and thereby decrease global myocardial workloads, as supported by our observations of the improved systolic function of remote left ventricular wall segments in treated animals and as previously postulated to be a mechanism of action underlying the efficacy of stem cell implant therapies.^5–14^

The dramatic decreases in fibrosis seen in treated animals in this and prior studies that appear to far exceed increases in the number of newly generated iCMs suggest that these observed reductions in fibrosis might also contribute to the significant improvements in EF observed in treated animals. Based on our preliminary data, it is conceivable that a paracrine effect of a relatively limited number of iCMs might underlie this reduction in fibrosis, due to the expression by iCMs of chemokines such as basic fibroblast growth factor and tissue inhibitor of matrix metalloproteinase–2 that have been reported to limit or reduce fibrosis.^43–45^

Alternatively, it is conceivable that administration of cellular reprogramming and/or VEGF transgenes diverts resident/scar fibroblasts away from their normal postinfarct differentiation into myofibroblasts, known to produce fibrosis *via* expression of collagen and other extracellular matrix components, and toward a more benign fate as iCMs.^43–50^ The 4‐fold reduction of myofibroblast populations we observed in GMT‐treated animals vs controls, consistent with a similar trend in reduced extent of fibrosis, supports this supposition. Theoretically, alternative processes such as myofibroblast apoptosis and/or repressed function (ie, decreased extracellular matrix component expression) could also play a role in such mechanisms.^43–50^

The use in the current study of lentivirus and adenovirus vectors that infect dividing and nondividing cells (including cardiomyocytes) raises the possibility also of GMT/VEGF effects on resident cardiomyocytes in addition to the targeting of fibroblasts in several prior studies.^30–34^ Some evidence in the literature suggests that such effects might include changes in cardiomyocyte structure, function, or stability/resistance to ischemia (ie, cardiac “super cells”).^43–50^ GMT and/or VEGF when administered via these vectors might influence the differentiation of other nonproliferating cells, such as resident cardiac progenitor cells, fibroblasts, or endocardial cells toward a cardiomyocyte fate and/or away from a myofibroblast phenotype.

We and others have previously demonstrated that scar vascularization is important to support the survival and function of stem cell implants, and we have delayed GMT administration for 21 days after AdVEGF‐All6A^+^ in accordance with our prior studies demonstrating a plateau in neovascularization beginning 3 weeks after the administration of VEGF.^40–42^ Preliminary cell survival studies that we have conducted likewise suggest that similar considerations would apply to observation of increased neovascularization providing the nutrient perfusion needed to support the conversion of low metabolic fibroblasts into high metabolic iCMs.

The importance of scar prevascularization to the presently described in situ cellular reprogramming strategy is supported by our observation of the ability of AdVEGF to induce scar vascularization in the setting of the acute myocardial infarction model used in the current study, together with our observation of significant improvements in EF when VEGF was administered as a supplement to GMT compared with the administration of GMT without VEGF. In the context of the lag in neovascularization induced by VEGF relative to the more rapid time course of myocardial infarction, and our observations of an equivalent extent of fibrosis in animals treated with vs without VEGF, the (comparatively limited) improvement in EF observed after VEGF administration (without GMT) is potentially attributable to the antiapoptotic properties of VEGF, as well as its angiogenic properties in promoting the viability and/or function of border zone cells.^43–50^

### Study Limitations

One significant limitation of the current study is that our use of a rat model as opposed to a mouse model precludes our readily accessing transgenic techniques to facilitate “cell of origin” studies. The limited visualization of GFP markers that we experienced at the extended, 7‐week end point of these studies further impaired these investigations. Given prior studies confirming scar fibroblasts as the source of iCMs in vivo, we believe that the advance of this work to a larger animal model and a second species outweighs the disadvantages of not being able to generate these otherwise duplicative data.^28–35^ This limitation is also mitigated by our demonstration of differences between groups in cardiomyocyte and myofibroblast marker positive cells, which has provided new insights into potential mechanisms of actions.

A second limitation of this and prior cellular reprogramming studies is the relatively inefficient transdifferentiation rate of fibroblasts into iCMs. Although Song et al appear to have made progress in addressing this challenge by their addition of a fourth reprograming gene, *HAND2*, to their treatment “cocktail,” the need to “hit” target cells with 4 different gene transfer vectors remains a limitation of current vector strategy.^32–34^ Alternative “triple transgene” or other vector constructs and/or microRNA strategies as demonstrated by Jayawardena et al could potentially address this challenge and further enhance the efficacy of this proposed new therapy.^34,51–52^

Finally, it is conceivable that delayed administration of either the adenovirus or lentivirus vectors might likewise yield greater effect if this administration was delayed to allow amelioration of the immediate postinfarct inflammatory milieu.^52^ Unfortunately, this strategy would be a prohibitive technical challenge in the current small animal model as it would involve 3 operations. Testing of this hypothesis in additional studies at extended time points to ensure that transdifferentiation remains stable is planned in a larger animal model now that these “proof of principle” data have been established.

## Conclusions

In the context of these limitations, the present study advances prior cellular reprogramming investigations by now incorporating angiogenic pretreatment of the infarcted myocardium as a means of enhancing a new, potentially clinically meaningful strategy to improve the function of postinfarct myocardium. Whereas the mechanisms underlying these outcomes are still not fully elucidated, it would appear that iCM generation from scar fibroblasts in situ likely contributes to this process.
